# Utilizing 3D printing to assist pre-procedure planning of transjugular intrahepatic portosystemic shunt (TIPS) procedures: a pilot study

**DOI:** 10.1186/s41205-023-00176-w

**Published:** 2023-04-13

**Authors:** Lucas Richards, Shiv Dalla, Sharon Fitzgerald, Carissa Walter, Ryan Ash, Kirk Miller, Adam Alli, Aaron Rohr

**Affiliations:** 1 University of Kansas School of Medicine, 3901 Rainbow Boulevard, 66160 Kansas City, KS USA; 2 Department of Population Health, University of Kansas Medical Center, 3901 Rainbow Boulevard, Mail Stop 1008, 66160 Kansas City, KS USA; 3 Department of Radiology, University of Kansas Medical Center, 3901 Rainbow Boulevard, Mail Stop 4032, 66160 Kansas City, KS USA

**Keywords:** 3D printing, TIPS, Procedure planning, Anatomic segmentation, Pilot study.

## Abstract

**Background:**

3D (three-dimensional) printing has been adopted by the medical community in several ways, procedure planning being one example. This application of technology has been adopted by several subspecialties including interventional radiology, however the planning of transjugular intrahepatic portosystemic shunt (TIPS) placement has not yet been described. The impact of a 3D printed model on procedural measures such as procedure time, radiation exposure, intravascular contrast dosage, fluoroscopy time, and provider confidence has also not been reported.

**Methods:**

This pilot study utilized a quasi-experimental design including patients who underwent TIPS. For the control group, retrospective data was collected on patients who received a TIPS prior to Oct 1, 2020. For the experimental group, patient-specific 3D printed models were integrated in the care of patients that received TIPS between Oct 1, 2020 and April 15, 2021. Data was collected on patient demographics and procedural measures. The interventionalists were surveyed on their confidence level and model usage following each procedure in the experimental group.

**Results:**

3D printed models were created for six TIPS. Procedure time (p = 0.93), fluoroscopy time (p = 0.26), and intravascular contrast dosage (p = 0.75) did not have significant difference between groups. Mean radiation exposure was 808.8 mGy in the group with a model compared to 1731.7 mGy without, however this was also not statistically significant (p = 0.09). Out of 11 survey responses from interventionists, 10 reported “increased” or “significantly increased” confidence after reviewing the 3D printed model and all responded that the models were a valuable tool for trainees.

**Conclusions:**

3D printed models of patient anatomy can consistently be made using consumer-level, desktop 3D printing technology. This study was not adequately powered to measure the impact that including 3D printed models in the planning of TIPS procedures may have on procedural measures. The majority of interventionists reported that patient-specific models were valuable tools for teaching trainees and that confidence levels increased as a result of model inclusion in procedure planning.

## Background

Three-Dimensional (3D) printing is a manufacturing process that has recently been adopted by the surgical community in a variety of applications, such as the creation of patient specific anatomic models for use in preoperative planning and as intraoperative references [1–13]. The use of these anatomic models during the planning of surgical procedures has been shown to consistently lead to changes in operative plans and increased confidence in clinical decision-making [3–6]. This could be particularly useful in planning of procedures within interventional radiology where many of the risks involved are directly related to fluoroscopic use in difficult patient anatomy.

A patient-specific 3D printed model from pre-procedural imaging displays diagnostic information in a novel way, potentially improving the interventionist’s understanding of the patient’s anatomy and reducing the procedural fluoroscopy time. As a result, radiation exposure, procedure time, and contrast dosage may be reduced, minimizing the potential negative impact that the procedure may have on a patient. 3D printed models have also allowed for the selection and testing of specific wires and catheters before entering the procedure room, reducing material waste and cost [7–9]. This too improves patient safety, as the exchange of wires and/or catheters introduces new risks.

The integration of 3D printed models in procedure planning has been the subject of publications claiming that this technology facilitated better surgical outcomes in multiple different surgical specialties [3–13]. Moreover, multiple publications have demonstrated dimensional and anatomic accuracy of these models for surgical planning [14, 15]. There are few examples showing the promise of using 3D printed models to plan procedures in interventional radiology, but there is no current literature that quantifies the impact of this technique as a measurable procedural outcome [8, 16]. Moreover, there is not sufficient clinical assessment of its efficacy via physician input (i.e. surveys). The application of this technology may have an over-arching impact on the outcomes of interventional radiology procedures, and there is a clear need for more studies to demonstrate and quantify its value so it may potentially become a reimbursable part of endovascular intervention [3, 7]. The purpose of this pilot study was to test the feasibility of the described model creation process. Additionally, this study set out to assess the impact that the inclusion of a 3D printed anatomic model in planning transjugular intrahepatic portosystemic shunt (TIPS) procedures has on the outcomes of procedure time, radiation exposure, intravascular contrast dosage, fluoroscopy time, and provider confidence.

## Methods

### Study design

This single-center pilot study utilized a quasi-experimental study design with a retrospective control and prospective experimental group, both including patients that underwent TIPS at a single institution. The patients in the control group had procedures that were planned and performed in a conventional manner without 3D printed models as part of procedure planning. The patients in the experimental group had procedures that included a 3D printed model that was manufactured and integrated into the procedure planning process.

### Participants and setting

Retrospective data was collected from patients that received TIPS between October 1, 2017 and October 1, 2020 and were included in the control group. Prospective data collection started on October 1, 2020 and a patient-specific 3D printed model was created for patients that underwent TIPS from October 1, 2020 to April 15, 2021 at the study institution. Factors including inadequate imaging quality and short lead time (in urgent/emergent situations) limited the ability to create a model for every TIPS procedure that was performed in the prospective period. Only the patients in which a model was successfully made and given to the interventionist prior to the procedure were included in the experimental group. Study procedures were approved by the Institutional Review Board at the study institution. Written informed consent was obtained from patients in the experimental group.

### Inclusion/exclusion criteria

Patients included in the control group underwent TIPS as the study institution between October 1, 2017 and October 1, 2020 and were at least 18 years of age. Patients included in the experimental group were at least 18 years of age, underwent a TIPS procedure at the study institution between October 1, 2020 and April 15, 2021, had a CT or MRI taken within one year prior to the scheduled procedure, had 48 h of lead time prior to the procedure to allow for model creation, and had a model created and given to the interventionist 24 h prior to the procedure start time.

### Model creation

**Fig. 1:**
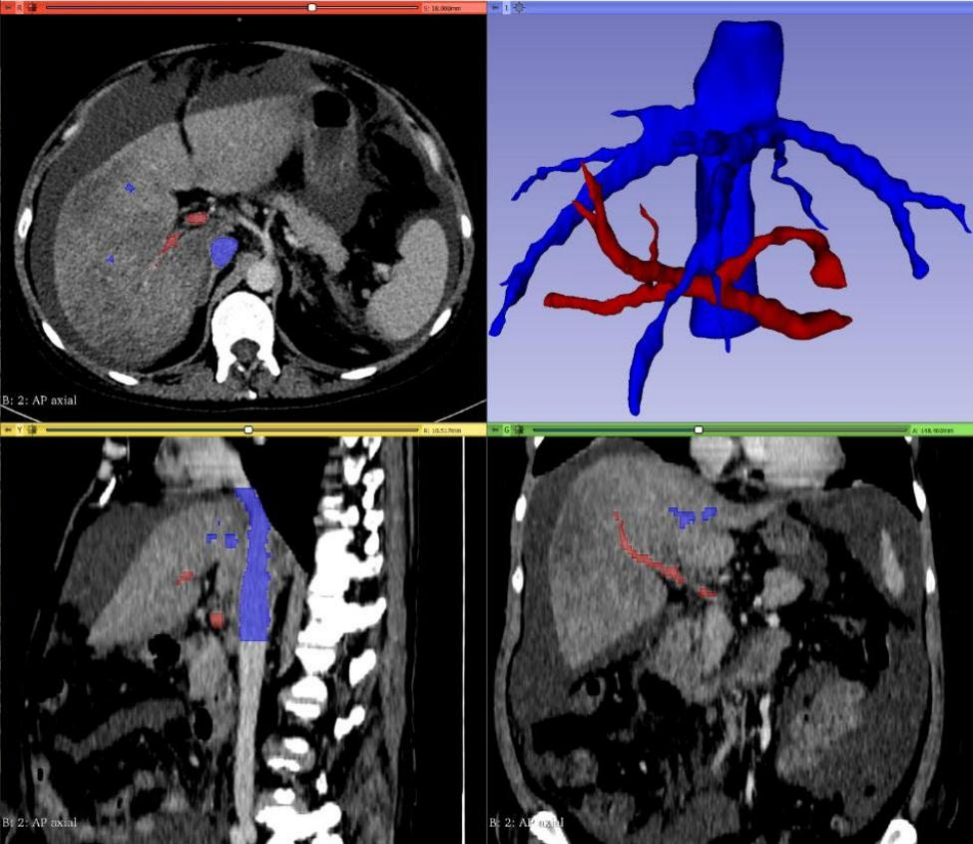
Segmentation Process A screen capture of the segmentation software 3D Slicer shows a model being created from a CT scan. On the top left, bottom right, and bottom left, different views of the patient’s imaging are displayed with bright colors overlaying the anatomy that has been selected from the background CT (red = portal venous, blue = hepatic/systemic venous). On the top right the resulting 3D geometry is displayed

Patient-specific 3D printed models were created utilizing Computed Tomography (CT) imaging that was obtained during routine patient care. The anatomy to be modeled was determined by the interventionist scheduled to perform the procedure and communicated to the study team. This communication was done in person while both parties reviewed imaging to ensure accurate goals and expectations. Deidentified images were copied to discs. 3D segmentation of the desired anatomy was performed in 3D Slicer by study team members that were trained in this technique. A screenshot of this process is shown in Fig. 1. Segmentation time was not measured in this study but was approximately 2–4 h for each model. Poor quality imaging greatly increased the time spent segmenting anatomy.

A screen capture of the segmentation software 3D Slicer shows a model being created from a CT scan. On the top left, bottom right, and bottom left, different views of the patient’s imaging are displayed with bright colors overlaying the anatomy that has been selected from the background CT (red = portal venous, blue = hepatic/systemic venous). On the top right the resulting 3D geometry is displayed.

**Fig. 2:**
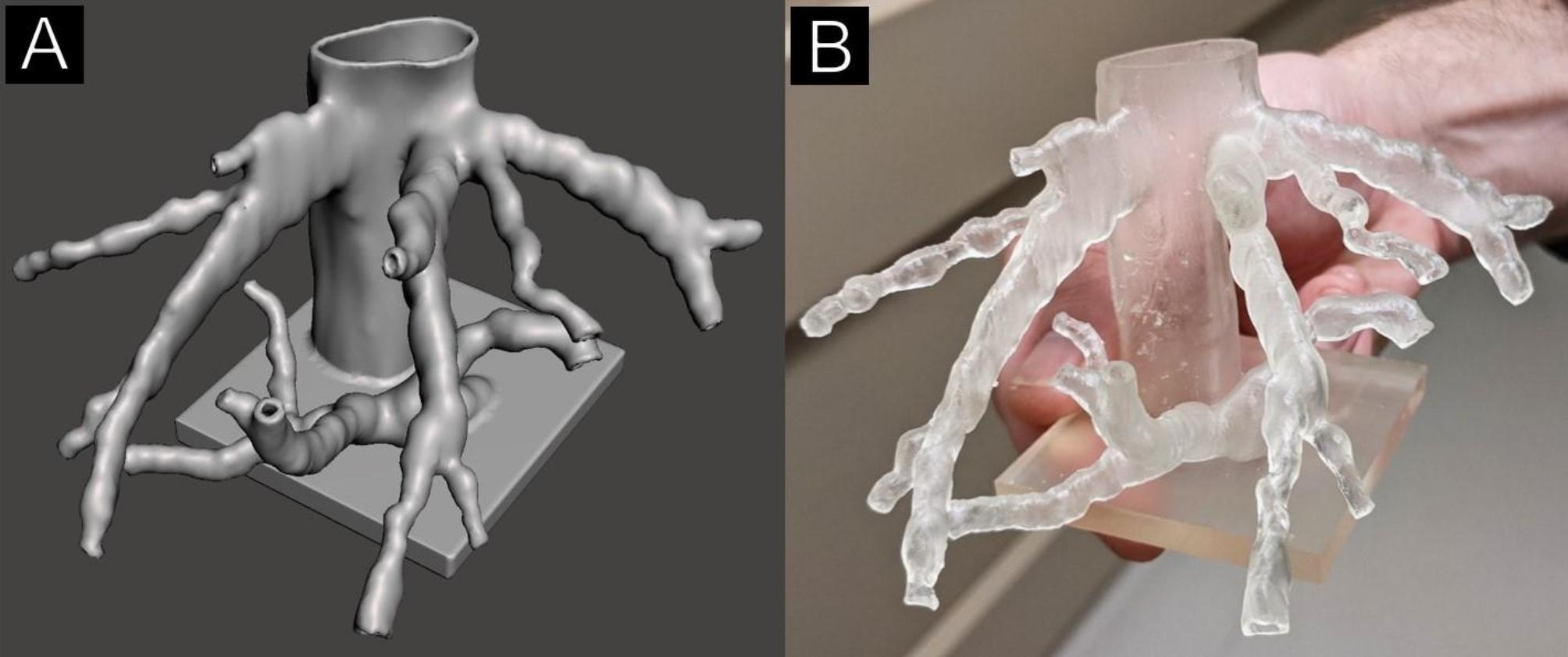
3D Model for TIPS planning (**A**) 3D rendering of a model displayed in Meshmixer. This is the final geometry sent to the printer but does not yet include the support structures required for a successful print. (**B**) The finished 3D model in the hands of an interventionist, now ready to be used in the procedure planning process

The models created using 3D Slicer were representations of the vessel lumens created by picking up the radiopaque intravascular contrast. However, the goal of the models was to display a representation of the patient’s anatomy so further processing was required. Using Meshmixer, the surfaces of the .STL files were extruded from, and the model was then made hollow in order to create 1.2 mm wall thickness tubular structures that had an inner surface geometry that matched the 3D model exported from 3D Slicer. The 1.2 mm wall thickness was chosen for this study as it allowed for a balance between strength and translucency in the resulting model. The fact that model wall thickness was not anatomically correct was communicated to the interventionists to avoid misunderstandings about true vessel wall thickness. In Fig. 2A, a model is displayed after the vessel walls have been extruded from the lumen. A base has also been created to allow the model to stand in anatomical orientation on a flat surface creating the final geometry that will be printed. The final print file was created in PreForm using a layer thickness of 0.1 mm, touchpoint size of 0.4 mm, density support setting of 0.8, and with internal supports disabled. Internal supports were used if absolutely necessary but were completely avoided in elements too small to allow removal in post-processing Printing was done using a FormLabs Form2 stereolithography printer with FormLabs Standard Clear resin. Print time averaged 8.9 h, with a standard deviation of 2.4 h. Average material cost per model was $12.81 US dollars with standard deviation of $5.98 US dollars. Material costs were considered linearly proportional to material volume which averaged 85.9mL with standard deviation of 40.2mL per model. The models were post-processed by washing with 99% isopropyl alcohol and curing with a FormLabs Form Cure. 3D printed models of the patient’s anatomy were given to the scheduled provider at least 24 h prior to the procedure start time. Interventionists were free to use the model as they saw fit and surveyed about how they chose to use the model in their planning process. A completed model is shown in Fig. 2B.

### Data collection and measures

Data collected in this study included demographics such as age, gender, race, and ethnicity. Procedural measures such as procedure time (min), fluoroscopy time (min), radiation exposure (mGy), and intravascular contrast dosage (ml) were captured during each procedure. All data were recorded as a normal part of documentation and were collected for analysis retrospectively from procedure notes in the electronic medical record in both the control and experimental groups. A participant was only included if all measures of interest were available.

**Fig. 3:**
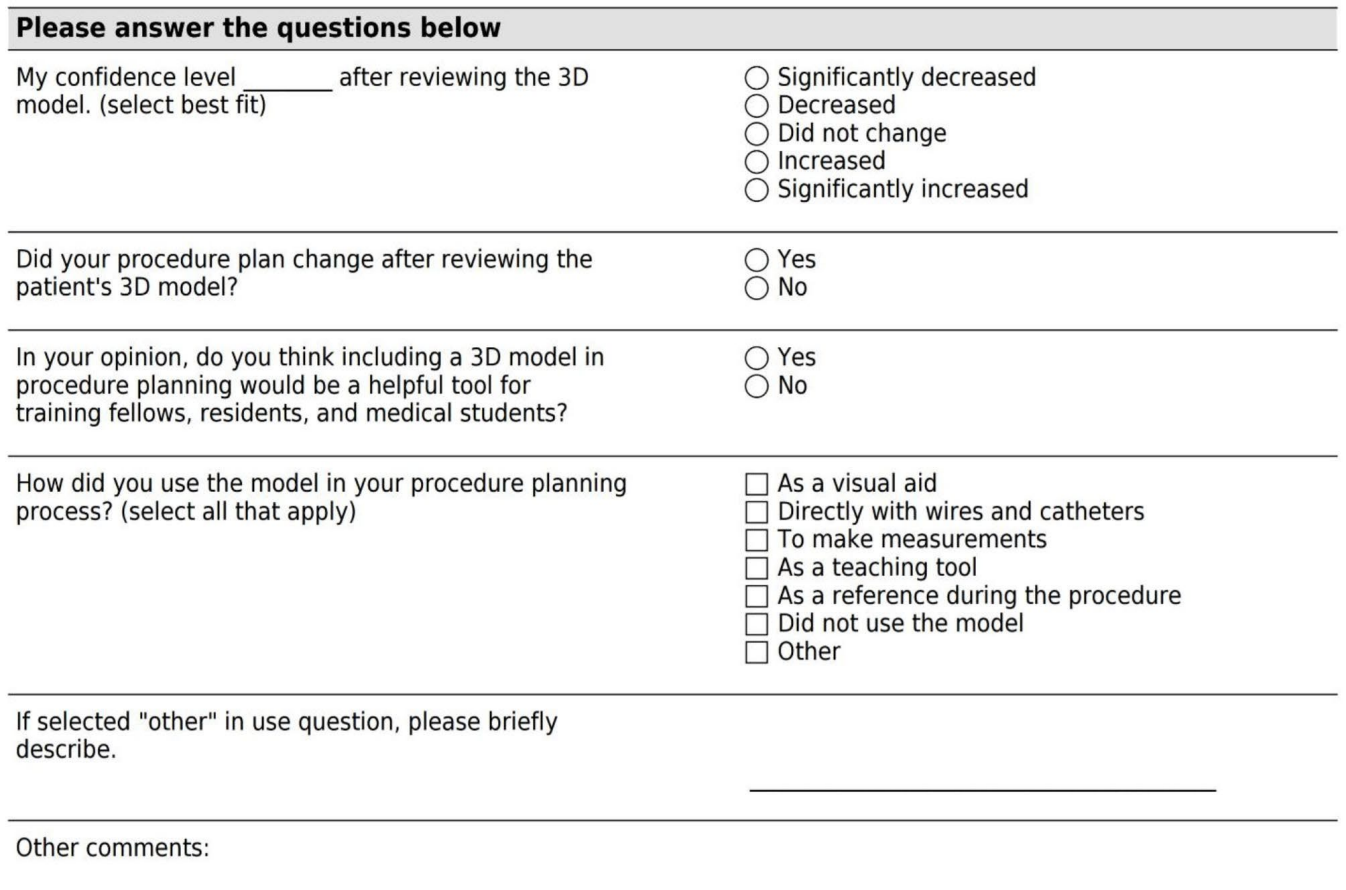
Post-Procedure Survey Interventionists were sent this short survey following each case that included a model in the planning process

A short survey was sent electronically immediately following each prospective procedure to the interventionists electronically. The questions asked about model usage and the impact the models had on interventionist confidence. The survey as it appeared to the interventionist is shown in Fig. 3.

### Data analysis

All data was stored securely in a REDCap database and deidentified when exported for analysis [17]. Analysis was carried out in SAS v9.4 with groups considered as a whole. An intention to treat analysis was employed. Descriptive statistics were used to examine population demographics. T-tests and chi-squared tests were used to make comparisons between the experimental and control groups.

## Results

A total of 26 participants were included in the study with 20 in the control group and 6 in the experimental group. Eleven TIPS were performed at the study institution during the prospective period, three had insufficient imaging for model creation, one had insufficient lead time for model creation, and one declined to enroll in the study. Differences in age (p = 0.38), gender (p = 0.51), race (p = 0.72), and ethnicity (p = 0.58) between the two groups weren’t statistically significant and were likely due to chance (See Table 1).

**Table 1:** Demographics

	Control n = 20	Experimental n = 6	p-value
**Age** Mean(SD)	63.1 (10.4)	58.5 (13)	0.38
**Gender**			0.51
Female n(%)	7 (35)	3 (50)	
Male n(%)	13 (65)	3 (50)	
**Race**			0.72
White n(%)	18 (90)	6 (100)	
Other n(%)	2 (10)	0 (0)	
**Ethnicity**			0.58
Hispanic n(%)	1 (5)	0 (0)	
Non-hispanic n(%)	19 (95)	6 (100)	

**Fig. 4:**
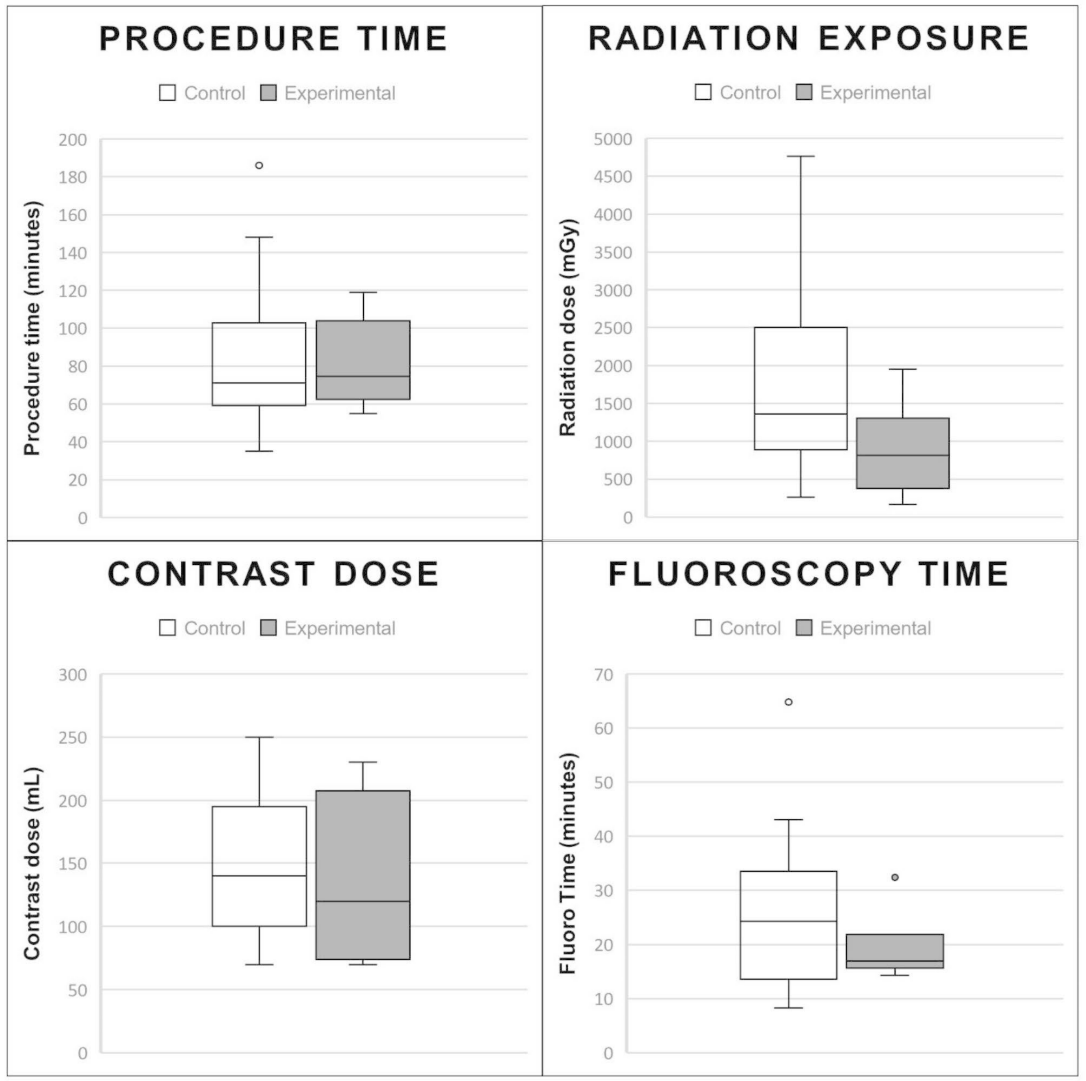
Procedural Measures

The most significant difference in procedural measures between the two groups was in radiation exposure, with a relative reduction of nearly 50% (p = 0.09). This however failed to reach the statistical significance threshold of p < 0.05. Mean fluoroscopy time (p = 0.26), mean intravascular contrast dosage (p = 0.75), and mean procedure time (p = 0.93) were also not significantly different between the two groups (See Table 2; Fig. 4).

**Table 2:** Procedural Measures

	Control		Experimental		p-value
	n = 20		n = 6		
	Mean (SD)	Median (Q1, Q3)	Mean (SD)	Median (Q1, Q3)	
Procedure time (min)	82.5 (38.1)	70.9 (59.7, 98.5)	81.2 (24)	74.5 (65.8, 94.5)	0.93
Radiation (mGy)	1731.7 (1133.9)	1358.5 (899, 2461.5)	880.8 (616.5)	814.5 (522.2, 1039.8)	0.09
Contrast (ml)	143.8 (49.3)	140 (100, 185)	135.8 (65.9)	120 (83.8, 182.5)	0.75
Fluoroscopy time (min)	25.7 (13.4)	24.3 (15.1, 32.9)	19.2 (6.6)	16.9 (16.3, 18)	0.26
p-value reported from t-test					

### Survey data

The primary operator was a staff physician with experience ranging from 3 to 15 years post-training with an accompanying trainee. A total of 13 post-procedure surveys were sent to these interventionists during prospective data collection and 11 responses were received. In response to the question “Did your procedure plan change after reviewing the patient’s 3D model?”, 5 (45.5%) chose “Yes.” In response to the question “In your opinion, do you think including a 3D model in procedure planning would be a helpful tool for training fellows, residents, and medical students?”, all 11 (100%) chose “Yes.” When asked to examine how the 3D model affected their confidence level, 10 (90.9%) responded with either “increased” or “significantly increased.” In response to the question “How did you use the model in your procedure planning process?”, the most popular response was “As a visual aid” with 11 (100%) responses, followed by “As a teaching tool” with 8 (72.7%) responses, “During the procedure” with 5 (45.5%) responses, and “Making measurements” with 3 (27.3%) responses. No responses were received that indicated the model was not used in the planning process.

## Discussion

### Interpretation of results

Our study was not sufficiently powered to detect statistically significant differences in procedural measures between the control and experimental groups due to a limited data collection period and nature of a pilot study.

Responses to post-procedure surveys from the interventionists were positive. Over 90% of the responses reported that confidence was either increased or significantly increased. Being able to look at and manipulate a 3D model of the anatomy prior to the procedure seems to reduce guess work and allow for more confidence in the procedural plan. Every interventionist surveyed responded that a 3D model would be a helpful tool for training fellows, residents, and medical students. This supports the idea that a physical 3D representation, in addition to traditional two-dimensional imaging, aids in understanding patient specific anatomy.

Utilization of the models in the pre-procedure planning process was observed by study team members to better understand how these models were being used. One of the ways that the models were helpful was determining the angle and distance of “throw” needed toward the portal veins. Understanding the vector down which the needle should be aimed seemed to be easier on a physical 3D model compared to a 3D volume rendered image. A 3D rendering on a computer monitor, shown on Fig. 2A, is only being displayed in two dimensions, the third is still left up to interpretation. The 3D printed model, shown in Fig. 2B in the hands of an interventionist, requires less interpretation to understand the orientation of the hepatic vessels. Another way that these models helped interventionists was deciding which of the hepatic veins to access to have the highest probability of finding the portal system based on relative proximity. This study was not intended to capture this as an outcome, so study team observations remain anecdotal.

### Low-quality imaging

**Fig. 5:**
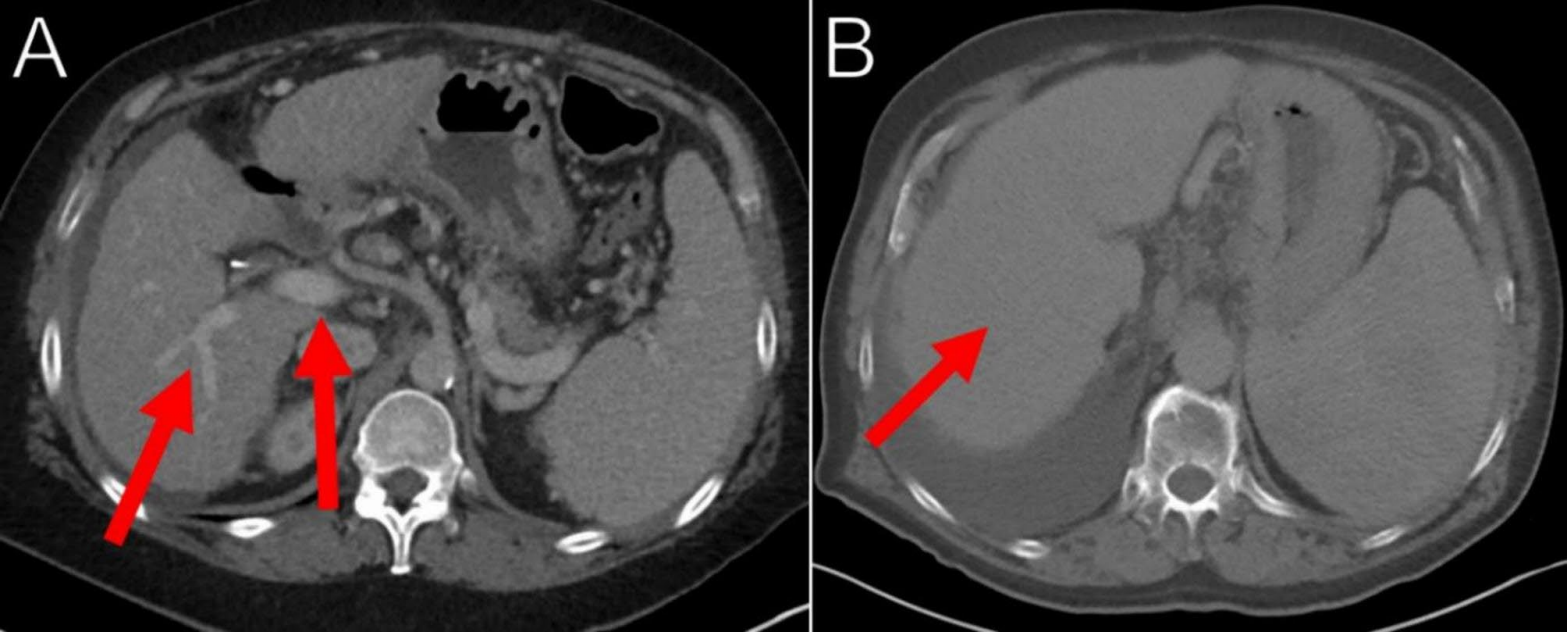
Variable Opacification of the Hepatic Vessels (**A**) Contrast enhanced CT in the portal venous phase displayed in the axial plane shows opacification of the hepatic vessels (arrows) making segmentation challenging but possible. (**B**) Contrast enhanced CT in the venous phase displayed in the axial plane demonstrates very poor opacification of the hepatic vessels (arrow) making segmentation and model creation impossible

Modeling for TIPS procedures proved challenging as the portal and hepatic venous systems were not always opacified well. This was primarily due to imaging being acquired at outside facilities utilizing imaging protocols different from the study institution. Faint vascular structures surrounded by noisy liver parenchyma often required manual “painting” of structures on each slice of imaging to adequately capture the anatomy. In 4 of the 10 attempted TIPS cases, the imaging of the liver vessels was so poor that a model was not able to be created at all. A CT with adequate contrast between hepatic vasculature and parenchyma is shown in Fig. 5A, but the vessels in Fig. 5B have no difference in density compared to the liver parenchyma, making selection impossible.

This resulted in fewer patients being included in the experimental group than were initially consented. More importantly this shows a limitation of this technology. Segmentation can be an augmentation to conventional imaging techniques but ultimately is only redisplaying the information that is already available in a different way. If the image acquisition is subpar for the desired application, segmentation cannot overcome this limitation.

### Experience with desktop stereolithography (SLA) printing

This experience has demonstrated the use of imaging that is already available in the procedure planning process to make models with the goal to aid interventionists. This required no additional time burden or health risk to the patient. Using the described approach, these models can be created in a little as 24 h once imaging is available and had an average material cost of $12.80 US dollars per model, negligible compared to that of a TIPS procedure. Material cost is not inclusive of other costs incurred when utilizing 3D printed models such as human resources and overhead which previous studies have described to be in excess of $2000 per model [18]. The printer used for the models in this study was a Formlabs Form2 SLA printer. Compared to other printers used for medical applications, the Form2 is affordable and user friendly, while maintaining consistency. No print failures were experienced during this study. This sample suggests that a high-cost printing system may not always be needed to produce 3D printed anatomic models for use in a medical setting. More advanced systems may have added functionality such as full-color and multi-material printing. We did not aim to utilize these features in our study, choosing to focus on accurate spatial demonstration with desktop-level printing hardware. Color printing may be useful in delineating portal vein from hepatic vein in the future.

### Personalized patient education

Several patients enrolled in the study were enthusiastic to be involved and asked to take their models home with them. In several cases, the models were used to educate the patient about the procedure they were about to undergo, and these patients expressed increased understanding of the procedure after seeing the model. This study was focused on procedural measures and the reactions of interventionists, but future studies should explore the impact this had on patients involved beyond their procedural measures.

### Limitations

While this study shows a consistent model creation method and trended towards reduction of radiation exposure, it was underpowered to detect statistically significant differences in outcomes between the experimental and control groups. A study including more participants is needed to further explore the impact of these models on procedural measures.

The non-concurrent method of data collection for the experimental and control groups could introduce important variables that could not be controlled for including but not limited to operating physicians, interventionist experience, and possible changes in hardware used for endovascular access and imaging. This should be addressed in future study design changes.

A limitation of using 3D printed models for procedural planning in the foreseeable future is the time required to make a model. While the turn-around time of 24 h for a model was quicker than expected, this limits its applications in emergent and most urgent situations.

## Conclusions

This pilot study demonstrates 3D printing can be used to create patient-specific anatomic models for procedure planning in interventional radiology without additional image collection. This can be done at a low cost and with no added risk to the patient’s health. This study wasn’t sufficiently powered to detect differences in procedural outcomes when including a 3D printed model in pre-procedural planning of TIPS procedures. There is a need for further study of this application of technology to assess its impact on procedural measures. Regardless, 3D printed models consistently improved the confidence level of interventionists, were beneficial to trainees, and in some instances led to the changing of procedural plans in this study. The addition of a 3D printed model as a resource in procedure planning may improve patient care. Access to this technology should be expanded to interventionists in a meaningful way, with the goal of maximizing preparedness and as a result, patient safety.

## Data Availability

The datasets used and/or analyzed during the current study are available from the corresponding author on reasonable request.

## Abbreviations

3D: Three-dimensional
TIPS: Transjugular intrahepatic portosystemic shunt
CT: Computed tomography
SLA: Stereolithography
SD: Standard Deviation
USD: United States Dollar
